# Epidemiology and antimicrobial susceptibility of *Vibrio cholerae* in recurrent cholera outbreaks in Ethiopia: a multicenter study

**DOI:** 10.1016/j.ijregi.2026.101021

**Published:** 2026-09-24

**Authors:** Zeleke Ayenew, Alem Abrha Kalayu, Rajiha Abubeker Ibrahim, Yeshambel Worku Demlie, Michael Getie, Negga Asamene Abera, Gemechu Tadesse Leta, Chalie Mulu Belete, Getachew Tollera Eticha, Aklilu Feleke Haile, Tadesse Eguale

**Affiliations:** a Aklilu Lemma Institute of Health Research, Addis Ababa University, Addis Ababa, Ethiopia; b Department of Microbiology, Immunology, and Parasitology, Addis Ababa, Ethiopia; c Ethiopian Public Health Institute, Addis Ababa, Ethiopia; d Department of Microbiology, Amhara Public Health Institute, Bahir Dar, Ethiopia; e Ohio State Global One Health, Addis Ababa, Ethiopia

**Keywords:** Recurrent cholera, *Vibrio cholerae*, Biotype, Serotype, Antimicrobial resistance

## Abstract

**Objectives:**

The recurrence of cholera continues to be a public health concern in Ethiopia. The emergence of antimicrobial-resistant *Vibrio cholerae* strains has complicated case management. Thus, this study aimed to assess the epidemiologic distribution and antimicrobial susceptibility profile of *V. cholerae* in Ethiopia.

**Methods:**

A cross-sectional study was conducted among patients in cholera treatment centers and health care facilities in Ethiopia from September 2023 to September 2025. Diarrheic stool and suspected water samples were collected and transported to the Ethiopian Public Health Institute to isolate and confirm *V. cholerae* using standard microbiology techniques. Antimicrobial susceptibility testing was performed by the Kirby-Bauer disc diffusion and E-test methods in accordance with updated Clinical and Laboratory Standards Institute guidelines.

**Results:**

Among 356 stool samples and 30 water samples, 66.3% and 3.3% were culture-positive for *V. cholerae*, respectively. All isolates were confirmed as *V. cholerae* O1 serotype Ogawa across all regions. The isolates were 100% susceptible to chloramphenicol and meropenem, 99.6% to tetracycline and 99.2% to ciprofloxacin but all isolates were resistant to azithromycin 98.7% to ampicillin and trimethoprim-sulfamethoxazole. Nearly all isolates were multidrug-resistant (99.6%).

**Conclusion:**

*V. cholerae* O1 Ogawa was the only serotype circulating in a long-standing cholera outbreak in Ethiopia, with a high prevalence of multi-drug resistant. A high rate of azithromycin resistance highlights the urgent need to revise national cholera treatment guidelines.

## Highlights

• A closely related strain of *Vibrio cholerae* O1 serotype Ogawa is circulating across multiple regions of Ethiopia during a prolonged cholera outbreak.
• Multidrug-resistant *V. cholerae* isolates were highly prevalent, accounting for 99.6% of all isolates in the current study.
• A high level of azithromycin resistance was observed, raising concerns about its continued use as an empirical treatment option in clinical settings.

## Introduction

Cholera is a severe acute watery diarrheal disease that can kill within hours if left untreated. Worldwide, cholera causes an estimated 1.3-4.0 million cases and 21,000-143,000 deaths annually [1]. According to recent global epidemiologic data, there were 535,321 cases and 4007 deaths in 2023 [2], with a rise to 560,823 cases and 6028 deaths in 2024 [3] across 60 countries. By 2025 [4], there were 614,828 cases and 7598 deaths. In Ethiopia, over 66,898 cases and 829 deaths, corresponding to an average case fatality rate of 1.21%, were recorded since the onset of the outbreak in 2022 [5].

The conducive circumstances for cholera outbreaks in numerous locations worldwide include displacement brought on by armed conflicts, natural disasters, and drastic climate change [6]. Recently, Sub-Saharan Africa, Southeast Asia, and the Middle East reported several cholera outbreaks, mainly due to war situations, humanitarian and refugee crises, and natural disasters such as earthquakes, leading to the displacement of populations, overcrowding, and disruption of health care facilities and basic hygiene [7].

The main causative agent of cholera is a bacterium known as *Vibrio cholerae*, a Gram-negative bacillus having more than 200 different serogroups. Toxigenic (cholera toxin-producing) strains of *V. cholerae,* belonging to the two major serogroups O1 and O139, are responsible for most cholera outbreaks. The O1 serogroup is further classified into two phenotypically distinct biotypes, classical and El Tor. Both biotypes can be subdivided into three serotypes: Ogawa, Inaba, and, very rarely, Hikojjima [8].

The O1 serogroup El Tor biotype strains are associated with a higher incidence of asymptomatic infections and rapid spread, although they are also capable of causing severe disease [9]. *V. cholerae* O139, on the other hand, is not subdivided into biotypes or serotypes. Rather, it is a distinct serogroup that emerged from the El Tor lineage, characterized by a unique O antigen and polysaccharide capsule, which initially enabled it to evade preexisting immunity to O1 and cause extensive epidemics after its emergence in the Indian subcontinent in 1992. Despite its similar virulence, O139 did not become established as the dominant pandemic lineage. Its incidence declined markedly after the mid-1990s due to increased population immunity against O139, and it is now reported only sporadically, primarily in parts of South Asia [10].

Controlling cholera and reducing mortality rates in a humanitarian setting require a multimodal strategy that combines treatment; social mobilization; quick surveillance; oral cholera vaccines; and improved water, sanitation, and hygiene [11].

Antimicrobial resistance can arise from the gradual accumulation of chromosomal mutations or through the horizontal gene transfer mediated by mobile genetic elements such as plasmids, integrons, and transposons. Widespread antibiotic use, particularly, mass administration of antimicrobials for prophylaxis in asymptomatic populations, is a likely risk factor for the selection of antimicrobial-resistant populations of bacteria [12]. The use of antibiotics as prophylaxis for home contacts of patients with cholera during earlier epidemics in Ethiopia was shown to be associated with the increased antibiotic-resistant cholera strains [13]. However, there is limited evidence regarding the antimicrobial susceptibility status of circulating *V. cholerae* isolates, which have the potential to complicate case management. Therefore, the current study aimed to determine the epidemiology and antimicrobial susceptibility profile of *V. cholerae* in recurrent cholera outbreaks in Ethiopia.

## Materials and methods

### Study design and settings

A cross-sectional study was conducted from September 2023 to September 2025 among patients suspected to have cholera in Ethiopia. Cholera hotspot areas were identified based on case-reporting sites using data obtained from the Ethiopian Public Health Emergency and Management of Cholera report database. The selected hotspot areas were drawn from high case-reporting regions of the country, including locations that were also impacted by the presence of internally displaced persons and refugees residing in camps among the host population. In this study, the identified hotspot areas included Addis Ababa, Oromia, Amhara, Dire Dawa, and the Somali regions. A smaller number of samples were also collected from the Benishangul-Gumuz and Tigray regions (Figure 1).

**Figure 1:**
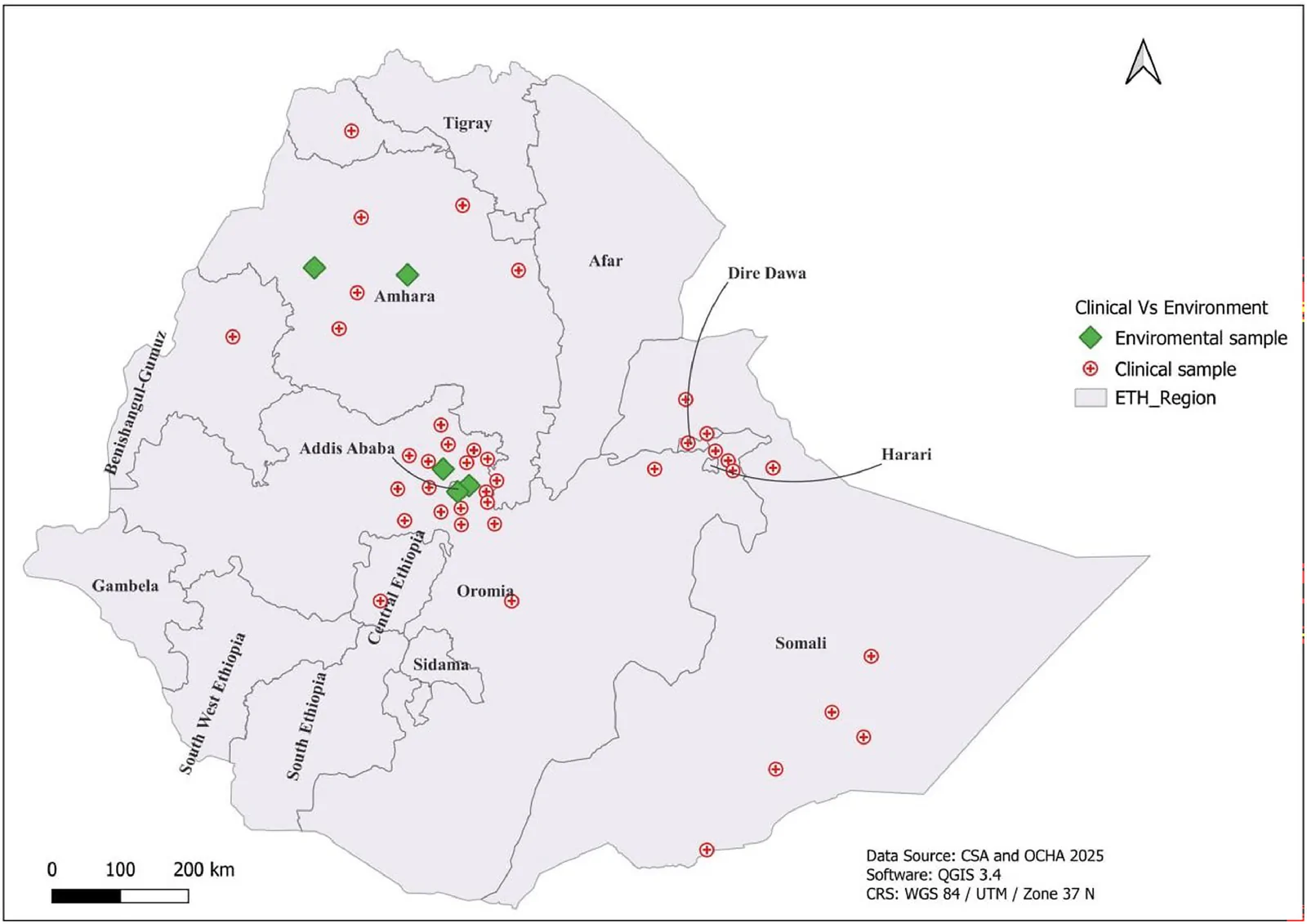
Epidemiologic distribution of clinical and environmental samples collected from different cholera hot spot sites across Ethiopia. Encircled red stars represent clinical sample areas distribution, and green boxes represent water samples collected from the highly suspected areas. Abbreviations: CSA, Central Statistical Agency; OCHA, United Nations Office for the Coordination of Humanitarian Affairs; CRS, coordinate reference system; WGS, World Geodetic System; UTM, Universal Transverse Mercator.

### Study population and sampling technique

The study population was patients diagnosed with symptoms of cholera in various cholera treatment centers (CTCs) during outbreaks in cholera hotspot areas. A purposive sampling technique was used to enroll the participants who fulfill the inclusion criteria. The sample was collected consecutively from inpatients admitted for cholera treatment and outpatients suspected of cholera cases until the required sample size was achieved.

### Inclusion and exclusion criteria

All patients in any age group who fulfilled the cholera case definition and had profound diarrhea were included. Patients who did not show typical symptoms of cholera and those who refused to participate or provide informed consent were excluded.

### Data collection

Patient information, including age, sex, residence, and clinical information, was collected by interviewing the patient or their guardians using a semi-structured questionnaire, a case investigation form, and a laboratory sample collection and analysis form.

### Clinical sample collection and processing

A total of 356 stool specimens were collected: 225 samples from the Amhara region, 64 from the Somali region, 32 from the Addis Ababa City Administration, 12 from the Dire Dawa City Administration, 11 from the Oromia Region, seven from Benishangul Gumz, and five from the Tigray region. Of the samples collected from the Amhara regional states, 177 samples were collected from Bahir Dar City, 23 from South Gondar, and 16 from North Gojjam. Among samples from Somali regional states, 16 were from Fafan, 15 from Shebelle, and six each from Sitti, Doobay, and Jijiga; four were from Mayaa-muluqo/Erer; three each were from Afder Zone, Gursum woreda, and Qabridahare; and one each was from Gode Zone and Bombas town. In addition, from Addis Ababa, stool specimens were collected from several sub-cities, including six from Yeka, five from Addis Ketema, four from Kolfe Keranyo, three from Arada, and six from Lemi Kura during the cholera outbreak (Figure 1).

A fresh diarrheic stool sample (3-5 mL) was collected from each suspected individual. A sterile fecal swab was dipped into each sample and transferred to Cary Blair transport medium. Then, the samples were transported to the National Clinical Bacteriology and Mycology Reference Laboratory using a triple packing system at ambient temperature. Upon receiving, each swab sample was inoculated into 5-7 mL alkaline peptone water and incubated at 37°C for 6-8 hours. Then, a 10-µL loopful of the suspension was taken from the surface and topmost area of the broth, where *V. cholerae* is preferentially expected to grow. It was sub-cultured on thiosulfate citrate bile salt (TCBS) agar and incubated aerobically at 37°C for 16-18 hours. A pure colony from TCBS was sub-cultured on 5% sheep blood agar (BAP) for biochemical tests, serotype, and antimicrobial susceptibility testing.

### Environmental water sample collection and processing

Water samples were collected from areas nearby patients with cholera. A total of 30 highly suspected of contaminated water samples were collected from various sources including; tap water (n = 24), drinking water from pipe (n = 3), spring water (n = 2), groundwater (n = 1), holy water (n = 1), and water stored in a tanker (n = 1) were bacteriologically processed for culture. Of these, 27 samples were collected from Addis Ababa and three from the Amhara region (Figure 1).

Heat-resistant water collection bottles were sterilized by autoclaving at 121°C for 15 minutes before use. Using a measuring flask, 250 mL of each water sample was filtered through a polycarbonate membrane filter with a pore size of 0.22 µm. The filter containing the retained bacteria was then aseptically transferred into a 50 mL centrifuge tube containing 12 mL of sterile 1 × phosphate-buffered saline (PBS). The tube was vortexed vigorously for 5 minutes to detach the bacteria from the membrane and to resuspend them in the PBS solution. Vigorous vortexing facilitates efficient bacterial detachment while minimizing cell lysis. A total of 1 mL of PBS suspension was added to an enrichment screw-capped flask containing 25-mL alkaline peptone water, and the mixture was incubated at 37°C. The next day, it was sub-cultured to TCBS and 5% BAP for identification [14]. The initial presumptive identification was based on detecting yellow colonies from the TCBS (Oxoid, UK) plate and beta-hemolytic colonies on BAP (Oxoid, UK).

### Biochemical analysis for identification of Vibrio cholerae

The following distinct biochemical tests were used to identify *V. cholerae* in presumed colonies from BAP: oxidase, string test (0.5% sodium deoxycholate), triple sugar iron agar for anaerobic carbohydrate fermentation, aerobic lactose fermentation, gas and H₂S production, lysine iron agar for lactose decarboxylase production, citrate, urea for urease production, and sulfide indole motility test [14].

### Antisera-based serotyping of Vibrio cholerae

Antisera testing was conducted to classify *V. cholerae* based on the type of specific O-antigens on its surface using antisera reagents (*V. cholerae* O1, Inaba; *V. cholerae* O1, Ogawa; *V. cholerae* O139, Bengal; and polyvalent [Inaba + Ogawa]) produced by Deben Diagnostic Ltd., a company in the United Kingdom. A pure colony of *V. cholerae* grown for 16-18 hours on a non-selective blood agar plate was used to perform the test. Initially, a single colony of *V. cholerae* was mixed with 0.85% physiological saline on a clean microscopic slide to create a homogenized suspension; then, a drop of each antiserum was applied to observe agglutination. The results were interpreted as positive when there was visible agglutination and negative when there was no visible agglutination, according to the manufacturer’s recommendation.

### Antimicrobial susceptibility testing

Antimicrobial susceptibility testing of the isolates was performed using the Kirby-Bauer disc diffusion method on Muller-Hinton agar (Oxoid, UK) in accordance with the updated Clinical and Laboratory Standards Institute (CLSI) M100 standard and M45 guideline. The recommended antibiotic discs were selected based on CLSI M45 guidelines against *V. cholerae* and included ampicillin (AMP) (10 µg), ceftazidime (30 µg), ceftriaxone (30 µg), azithromycin (AZM) (minimum inhibitory concentration [MIC]), chloramphenicol (30 µg), tetracycline (30 µg), trimethoprim-sulfamethoxazole (SXT) (1.25/23.75 µg), meropenem (10 µg), and polymyxin B (MIC). The MIC was determined using the E-test for AZM and ciprofloxacin [15]. *V. cholerae* isolates that were resistant to at least one antimicrobial agent in three or more classes of antimicrobials were considered multidrug-resistant (MDR) [16].

### Quality assurance

Before inoculation with a specimen, all media and tube biochemicals were prepared according to the manufacturer’s instructions, and their quality was assessed and recorded. The quality of TCBS media was checked for sterility by incubating uninoculated, freshly prepared media in an incubator for 24 hours; a growth promotion test was also performed using *Escherichia coli* American Type Culture Collection (ATCC) 25922 as a negative control and *V. cholerae* ATCC 16961 as a positive control. The quality of antibiotic discs was evaluated and verified initially and then switched to a routine weekly internal quality control system according to the clinical laboratory Standard Institute (CLSI) guideline.

### Statistical analysis

Data were checked for completeness and consistency in Microsoft Excel version 10 and exported to SPSS version 22 for analysis. Descriptive statistics, such as frequency, cross-tabulation, and proportion, were used to describe the sociodemographic characteristics of study participants. A bivariate logistic regression model was first fitted, and variables with *P* ≤ 0.02 were selected for inclusion in the multivariate logistic regression analysis. Adjusted odds ratios (AORs) with 95% confidence intervals (CIs) were calculated to estimate the strength of association between predictor variables and *V. cholerae* infection. A *P* < 0.05 was considered statistically significant.

## Results

### Demographic characteristics of cholera suspect cases

A total of 356 stool samples were collected from patients with acute watery diarrhea during the study period across the country. Of these, 199 (56%) of them were male. The median age of study participants was 28 years (interquartile range 22-40). The majority of the participants were adults aged 25-34 years, constituting most suspected cases (26.1%), followed by those in the adolescent age group 15-24 years, accounting for 23.9%, whereas the most vulnerable age groups, children under 5 years old, together with school-aged children (aged 5-14 years), represent 15.5% of the cases. Most of the participants were from the Amhara region (63.2%, n = 225) and the Somali region (18%, n = 64). There were fewer cases from the Benishangul Gumz region (2%, n = 7) and Tigray region (1.4%, n = 5). The highest proportion of sampling of suspected cases was during 2024 (66%), and a clear seasonal trend was observed, with cases peaking during summer (rainy season), accounting for 52.2% of the cases (Table 1).

**Table 1:** Enrolled participants distribution from September 2023 to September 2025 in Ethiopia’s recurrent cholera outbreak.

Variables	Category	Frequency (%)
Sex	Male	199(55.9)
	Female	157(44.1)
Age group (years)	<5	21(5.9)
	5-14	34(9.6)
	15-24	85(23.9)
	25-34	93(26.1)
	35-44	55(15.4)
	45+	68(19.1)
Regional distribution	Addis Ababa	32(9.0)
	Amhara	225(63.2)
	Benishangul Gumz	7(2.0)
	Dire Dawa	12(3.4)
	Oromia	11(3.1)
	Somali	64(18.0)
	Tigray	5(1.4)
Admission status of the patients	Inpatient	181(50.8)
	Outpatient	175(49.2)
Year of specimen collection	2023	57(16.0)
	2024	235(66.0)
	2025	64(18.0)
Seasonality	December to February (2023-2025)	52(14.6)
	March to May (2024-2025)	34(9.6)
	June to August (2024-2025)	186(52.2)
	September to November (2023-2024)	84(23.6)

Nearly half of the cases (n = 175) were managed in an outpatient setting, and the other half were from the inpatient ward of hospitals’ admissions and CTCs. According to the clinical characteristics of the study participants, 28.9% exhibited no signs of dehydration, whereas 61.8% displayed mild dehydration and 9.3% exhibited severe dehydration. During the data collection time, all patients were alive. After interviewing the patients about their history of prophylactic antibiotic use, 253 (71%) reported having taken antibiotics for other illnesses.

### Occurrence and serotype distribution of Vibrio cholerae in patients suspected of having cholera and environmental water samples

Among 356 stool specimens collected from patients suspected to have cholera, 236 (66.3%) were culture-positive for *V. cholerae,* whereas one (3.3%) of 30 environmental water samples was positive for *V. cholerae* obtained from the *Tanka Gebriel* well in the Fogera district of South Gondar Zone in the Amhara region. The culture positivity rate of suspected cases between September 2023 and 2024 was 49.7%, whereas it was 16.6% between October 2024 and September 2025. A clear seasonal trend was observed; culture positivity was proportionally high during the rainy season, at 39% (n = 139) among the cases.

All isolates showed β-hemolysis on sheep blood agar and had high-level resistance to polymyxin B (MIC ≥256 µg/mL). These features are consistent with established phenotypic markers of El Tor strains. Serotyping of the isolates showed that 235 of the clinical isolates and one isolate from water samples agglutinated with *V. cholerae* O1 Ogawa–specific antisera, whereas one isolate from the Somali region did not agglutinate with Ogawa, Inaba, or Bengal O139 antisera. This isolate was reported as non-O1/non-O139. The majority of the clinical isolates and one isolate from contaminated water circulating in the current outbreak were of the Ogawa serotype (Table 2).

**Table 2:** Serogroup and serotype distribution among clinical (n = 236) and environmental water sample (n = 1) isolates from September 2023 to 2025.

Year	Clinical isolates						Water sample isolates					
	No. of isolate	Somatic O-antigen serotyping					No. of isolate	Somatic O-antigen serotyping				
		VC O1 Ogawa n (%)	VC O1 Inaba n (%)	VC O1 Hikojjima n (%)	VC O139 Bengal	VC non-O1/non-O139		VC O1 Ogawa n (%)	VC O1 Inaba n (%)	VC O1 Hikojjima n (%)	VC O139 Bengal	VC non-O1/non-O139
2023	34	34 (100)	0	0	0	0	0	0	0	0	0	0
2024	160	159 (99.4)	0	0	0	1 (0.6)	1	1 (100)	0	0	0	0
2025	42	42 (100)	0	0	0	0	0	0	0	0	0	0
Total	236	235 (99.6)	0	0	0	1 (0.4)	1	1 (100)	0	0	0	0

Abbreviation: VC, *Vibrio cholerae*.

### Factors associated with Vibrio cholerae positivity

In multivariable logistic regression analysis, regional variation was significantly associated with *V. cholerae* positivity (chi-square [χ²] = 17.6, *P* = 0.007); specifically, the Amhara region (AOR = 4.7; *P* = 0.007; 95% CI 1.5-16.7) and Somali region (AOR = 5.4, *P* = 0.001; 95% CI 1.9-15.0) showed that the odds of culture positivity among suspected cases were 4.7 times and 5.4 times higher, respectively, compared with the cases in the capital, Addis Ababa. Other regions were not significantly associated with the positivity of the cases.

The overall effect of seasonality was a statistically significant association (χ² = 12.4, *P* = 0.006) with cholera case positivity that varied across seasons. Despite a high proportion of *V. cholerae* detection during the summer season (June to August), there was no statistically significant association (AOR = 1.7; *P* = 0.11; 95% CI 0.8-3.4) with cholera-confirmed cases compared with the autumn season (September to November). On the other hand, the odds of cholera cases during the spring season, from March to May, were low compared with autumn (AOR = 0.3; *P* = 0.032; 95% CI 0.1-0.9).

The overall patients’ age group was not significantly associated with *V. cholerae* positivity (χ² = 8.1, *P* = 0.15). However, children aged under 5 years were less likely to be positive for *V. cholerae* (AOR = 0.22, 95% CI 0.07-0.6, *P* = 0.008) compared with those with age group ≥45 years *Vibrio cholerae* positivity was not significantly associated with admission status (*P* > 0.05). Similarly, sex difference was not significantly associated with *V. cholerae* positivity (Table 3).

**Table 3:** Association factors of *Vibrio cholerae* positivity from clinical specimens.

Variable	Category	No. of specimen	*V. cholerae O1*–positive n (%)	COR 95% CI	*P*-value	AOR 95% CI	*P-*value
Patient demographic and clinical features analysis							
Sex	Male	199	138 (69.3)	Ref		-	
	Female	157	98 (62.4)	1.3 (0.8-2.1)	0.170		
Age group (years)	<5	21	8 (38.1)	0.2 (0.1-0.7)	0.009	0.2 (0.07- 0.6)	0.008
	5-14	34	20 (58.8)	0.6 (0.2-1.4)	0.24	0.6 (0.2-1.6)	0.36
	15-24	85	58 (68.2)	0.9 (0.4-1.8)	0.75	0.5 (0.2-1.0)	0.06
	25-34	93	66 (70.9)	1.0 (0.5-2.0)	0.95	0.6 (0.3-1.4)	0.25
	35-44	55	36 (65.4)	0.7 (0.3-1.7)	0.54	0.6 (0.2-1.4)	0.23
	45+	68	48 (70.5)	Ref		Ref	
Admission status	Inpatient	181	112 (61.8)	0.7 (0.4-1.0)	0.074		
	Outpatient	175	124 (70.8)	Ref			
History of antibiotics therapy	Yes	253	162 (64.0)	0.6 (0.4-1.1)	0.15		
	No	103	91 (88.3)	Ref			
Dehydration	No dehydration	103	69 (66.9)	Ref			
	Moderate	220	148 (67.2)	1.0 (0.6-1.6)	0.96		
	Sever	33	19 (57.5)	0.6 (0.3.1.5)	0.32		
Treatment given	ORS	207	137 (66.1)	0.9 (0.4-2.1)	0.95		
	RLS	36	24 (66.7)	1.2 (0.2-5.3)	0.84		
	Antibiotics	10	7 (70.0)	Ref			
Spatiotemporal analysis							
Regional distribution	Addis Ababa	32	11 (34.3)	Ref			
	Amhara	225	163 (72.4)	5.0 (2.3-11.)	<0.0001	4.7 (1.5-16.	0.007
	Benishangul	7	4 (57.1)	1.4 (0.3-7.6)	0.67	2.9 (0.4-22.	0.28
	Dire Dawa	12	9 (75.0)	5.7 (1.3-25.6	0.02	4.9 (0.8-29.	0.08
	Oromia	11	3 (27.3)	0.7 (0.2-3.2)	0.66	0.6 (0.1-3.6)	0.57
	Somali	64	42 (65.6)	3.6 (1.5-8.9)	0.005	5.4 (1.9-15)	0.001
	Tigray	5	5 (100)	—–		—-	
Specimen collection (years)	2023/2024	267	177 (66.3)	1.1 (0.7-1.6)	0.52		
	2024/2025	89	59 (66.3)	Ref		-	
Seasonality	winter	52	29 (55.8)	0.6 (0.3-1.3)	0.26	1.4 (0.5-3.5)	0.48
	spring	34	13 (38.2)	0.3 (0.1-0.7)	0.008	0.3 (0.1-0.9)	0.032
	summer	186	139 (74.7)	1.5 (0.8-2.7)	0.12	1.7 (0.8-3.4)	0.11
	autumn	84	55 (65.5)		.	Ref	

Ethiopian seasons—autumn (September to November), winter/dry season (December to February), spring (March to May), and summer/rainy season (June to August).

Abbreviations: AOR, adjusted odds ratio, AWD, acute watery diarrhea, COR, crude odds ratio, CI, confidence interval, ORS, oral rehydration solution, Ref, Reference, RLS, Ringer lactate solutions.

### Antimicrobial susceptibility profile of the Vibrio cholerae isolates

The antimicrobial susceptibility testing of clinical *V. cholerae* isolates revealed that all 236 isolates were 100% susceptible to chloramphenicol and meropenem, 99.6% to tetracycline, and 99.2% to ciprofloxacin. However, isolates demonstrated 100% resistance to AZM and 98.7% to AMP and SXT. An isolate of *V. cholerae* from an environmental water sample showed a similar pattern of antimicrobial susceptibility to the clinical strains; it was susceptible to tetracycline, ciprofloxacin, and chloramphenicol but resistant to AMP, AZM, and SXT. In addition, it was susceptible to meropenem but showed resistance to ceftazidime and ceftriaxone in its antimicrobial susceptibility profile (Table 4).

**Table 4:** Antimicrobial susceptibility pattern of *Vibrio cholerae* isolates collected from 2023 to 2025.

Antimicrobial agents	Clinical isolates (n = 236)			Environmental isolate (n = 1)		
	No.(%)S	No.(%)I	No.(%)R	No.(%)S	No.(%)I	No.(%)R
Ampicillin	1 (0.4)	2 (0.8)	233 (98.7)	0	0	1 (100)
Chloramphenicol	236 (100)	0	0	1 (100)	0	0
Ciprofloxacin	234 (99.2)	1 (0.4)	1 (0.4)	1 (100)	0	0
SXT	2 (0.8)	1 (0.4)	233 (98.7)	0	0	1 (100)
Azithromycin	0	0	236 (100)	0	0	1 (100)
Tetracycline	235 (99.6)	0	1 (0.4)	1 (100)	0	0
Ceftazidime	0	0	236 (100)	0	0	1 (100)
Cefotaxime	0	0	236 (100)	0	0	1 (100)
Meropenem	236 (100)	0	0	1 (100)	0	0

Abbreviations: I, intermediate; R, resistant; S, susceptible; SXT, trimethoprim-sulfamethoxazole.

### Antimicrobial resistance pattern of Vibrio cholerae isolates

The clinical isolates collected in the current study exhibited three distinct antimicrobial resistance patterns. One isolate, obtained from a stool sample of a pediatric patient from the Somali region, was resistant only to AZM. The majority of isolates (99.2%) showed resistance to AMP, AZM, and SXT, representing resistance to three different antimicrobial classes. Similarly, a single isolate obtained from a water sample exhibited this same resistance pattern. In addition, one isolate from an adult patient from Dire Dawa demonstrated non-susceptibility to four antimicrobials—AMP, AZM, SXT, and ciprofloxacin—belonging to four different antimicrobial classes. Overall, 99.6% of the isolates were MDR (Table 5).

**Table 5:** Antimicrobial resistance patterns of *Vibrio cholerae* isolated from stool specimens of diarrheic patients and water samples.

Resistance type	Antimicrobial class	Antimicrobial agents	No. of resistant isolates (%)
Clinical isolates			
R1	Macrolides	AZM	1 (0.4)
R3	β-lactam, macrolides, folate pathway inhibitor	AMP/AZM/SXT	234 (99.2)
R4	β-lactam, macrolides, folate pathway inhibitor, fluoroquinolones	AMP/AZM/SXT/CIP	1 (0.4)
Isoate from water sample			
R3	β-lactam, macrolides, folate pathway inhibitor	AMP/AZM/SXT	1 (100)

Abbreviations: AMP, ampicillin, AZM, azithromycin, CIP, ciprofloxacin, CTX, cefotaxime, SXT, trimethoprim-sulfamethoxazole.

## Discussion

The recent cholera outbreaks that began in August 2022: the first case of cholera erupted in the Hanan district of the Bale zone in the Oromia Region and gradually spread virtually to all regions of the country; 66,898 cases and 829 deaths, with an average case fatality rate of 1.21%, were recorded by the end of 2025 [5].

In the current study, *V. cholerae* was found in 66.3% of suspected cases from clinical samples. This is similar to the 62.9% report in a previous study in low- and middle-income countries [17] and the 60.6% in Southern Ghana [18] but lower than the 71.3% reported from Malawi [19]. It is higher than the previous study in Ethiopia, which reported 30.1% [13]. Several factors may account for the observed variations, particularly, methodological differences in participant selection, timing of specimen collection in relation to the stage of illness, previous antibiotic use, severity of diarrhea, and the volume of the sample collected. In addition, variations in specimen transportation and storage conditions, such as delays in transport, use of inappropriate transport media, and failure to maintain optimal temperature, may further contribute to a low detection rate of *V. cholerae*, in which our samples were collected during an enhanced epidemic compared with earlier studies with a low incidence of cases. Unlike earlier research, which was primarily community-based, we included half of the participants who were patients from health care facilities.

Although we tested many suspicious water samples used by patients with cholera symptoms, only 3.3% was positive for *V. cholerae*. This is comparable with other studies. A systematic review and meta-analysis found that the global final point-of-drinking water *Vibrio* prevalence was 5.1%. Africa had the highest prevalence at 6.3%, followed by Asia at 4.8% [20]. However, our result was half that of a study from Nepal, where 6.7% of drinking water samples were positive for *V. cholerae* [21]. This low culture positivity could be due to dormancy stage of *V. cholerae* because of very cold and nutrient-poor conditions that make them viable but not culturable. At this point, rapid diagnostic test and molecular methods, such as polymerase chain reaction are more likely to be appropriate to detect in epidemic settings.

In the present study, the detection of *V. cholerae* from suspected patients varies significantly with the season*:* a high rate of detection was observed during the rainy season *(*June to August) (χ² = 12.4, *P* = 0.006) compared with the dry seasons of the year, in line with a previous report from sub-Saharan Africa [22]. In most parts of Ethiopia, summer is the time of year when it rains the most. This can result in significant floods, which can destroy the sewage system, causing contamination of drinking water.

In our study, all isolates were phenotypically classified as serogroup O1 and serotype Ogawa. All regions have similar circulating serotypes, which is consistent with recent studies from Ethiopia that reported the dominant serotype as Ogawa [11] and a 2017 cholera outbreak in Addis Ababa [23]; however, a study from northwest Ethiopia reported a dominant serotype as Inaba about 15 years ago [24]. This finding may be explained by cross-border transmission of strains from Sudan, where serotype Inaba has been reported to be dominant.

Most cholera outbreaks in neighboring countries reported the presence of Inaba and Ogawa serotypes or a switch in dominance between the two. Similar to our findings, the Ogawa serotype was predominantly reported in cholera outbreaks in Somalia during 2017-2018 [25] and Kenya in 2022 [26]. It was also concordant with reports from most West African countries, including Niger, Burkina Faso, and Togo [6]. In contrast to our study, Inaba was the predominant strain during outbreaks in Kenya from 2007 to 2010 [27], South Sudan in 2017 [28], and Uganda in 2017 [29].

There is limited evidence to show serotype switching among Ethiopian isolates in various waves of cholera outbreaks that have happened over time; however, in Haiti, there was a shift in serotype, with Ogawa being the most prevalent serotype in 2018-2019 and Inaba being the most prevalent serotype in 2015-2017 and 2020-2021 [30]. Genomic analyses demonstrate that serotype switching between Ogawa and Inaba is mediated by alteration of the *wbeT* gene, which encodes a methyltransferase function during methylation of the terminal perosamine residue of lipopolysaccharides in the O antigen during the Ogawa serotype, but mutation or alteration can result in the expression of Inaba serotypes. Herd immunity may act as selective pressure that influences the dominance of a particular serotype by inducing population-level immunity against previously circulating strains. This dynamic can contribute to shifts in serotype predominance during successive outbreaks of *V. cholerae.* Consequently, cholera epidemics may occur without necessarily reflecting the emergence of novel strains but rather changes in population immunity and circulating serotypes [31]. Recent evidence suggests that O1 antigen methylation in Ogawa strains enhances *V. cholerae* colonization, infectivity, and resistance to antimicrobial peptides [32]. Thus, the continued presence of the prevalent Ogawa serotype in Ethiopia may be a factor in the protracted cholera epidemics.

According to the national cholera treatment guidelines of Ethiopia, doxycycline is the first line of treatment, and, alternatively, tetracycline, azithromycin, and ciprofloxacin are the treatment modalities in severe cases [33]. However, in this study, azithromycin showed 100% resistance, which is comparable to 90.9% resistance reported from Kenya [25]. Studies from Yemen showed an increased trend of azithromycin resistance from 50% in 2017 to 100% in 2018-2024 [34,35]. In contrast, a low rate of resistance (29%) to azithromycin was reported from India [36], and 100% susceptibility to erythromycin was reported in Zambia’s 2024 cholera outbreak [37]. This difference may be attributed to variations in the extent of macrolide use because prolonged or excessive use for prophylaxis can create selective pressure on susceptible *V. cholerae* strains, increasing the population of resistant strains.

The current study found that 99.6% of the isolates were MDR *V. cholerae*, which is comparable to other findings from high cholera-reporting nations, such as Kenya (99%) [26], Yemen (99%) [38], and Bangladesh (93%) [39]. This can be ascribed to multiple contributing factors, including the overreliance on prophylactic antibiotics, the ability of *V. cholerae* to acquire resistant genes from other antimicrobial-resistant bacteria through horizontal gene transfer, poor sanitation and hygiene, and humanitarian crises and environmental changes. Consequently, cholera has remained endemic in many regions and continues to cause recurrent, long-standing outbreaks.

### Limitation of the study

This study faced some limitations due to intermittent wave-like shifts of the outbreaks among regions, and limited drinking water samples were considered, other than different environmental reservoirs of *V. cholerae* in all areas of the county. We were unable to interpret biotyping of isolates with only polymyxin B resistance and characteristics of β-hemolysis, which require additional tests such as Voges-Proskauer, phage typing, and chicken red blood cell agglutination. The whole genome sequencing was not included in the current study due to the unavailability of reagents and delays in their delivery.

## Conclusion

This finding demonstrated that the main circulating strain responsible for long-standing cholera outbreaks in Ethiopia was the *V. cholerae* O1 serotype Ogawa strain. The detection of this dominant serotype in different cholera hotspot regions of the country in clinical and contaminated water sources suggests the dissemination of phenotypically similar bacterial strains in cholera hotspot areas and beyond. A high rate of resistance to optional medication for empirical therapy in clinical settings, such as azithromycin, was observed, whereas chloramphenicol, ciprofloxacin, and tetracycline showed good performance in the *in vitro* antimicrobial susceptibility tests. Hence, there is an urgent need to revise the national treatment guideline for cholera. Further molecular characterization of the isolates is warranted to generate comprehensive insights into the recurrent outbreaks and to elucidate the clonal dissemination of *V. cholerae* across the country.

## Funding

The research was jointly funded by the Ethiopian Public Health Institute and Addis Ababa University. The funders were not involved in the design, data analysis, manuscript preparation, or the decision to publish.

## Ethical approval and consent to participate

The authors confirm that all methods and procedures used in this study adhered to the relevant institutional and national ethical guidelines. The ethical clearance for this study was obtained from the institutional review board (IRB) of the Ethiopian Public Health Institute (IRB protocol No. EPHI-IRB-576-2024) and the IRB of Aklilu Lemma Institute of Pathobiology, Addis Ababa University. Written official letters were submitted to regional public health institutions about the study, and, where appropriate, permission was secured. Each study participant was informed about the purpose, objectives, and their right to participate or not participate in study activities. The study was conducted in accordance with the principles of the Declaration of Helsinki. To ensure confidentiality, all data were anonymized and encrypted, including patient names and medical record numbers.

## CRediT authorship contribution statement

**Zeleke Ayenew:** Writing – review & editing, Writing – original draft, Visualization, Supervision, Software, Resources, Methodology, Investigation, Funding acquisition, Formal analysis, Data curation, Conceptualization. **Alem Abrha Kalayu:** Writing – review & editing, Visualization, Validation, Supervision, Resources, Formal analysis, Data curation, Conceptualization. **Rajiha Abubeker Ibrahim:** Writing – review & editing, Supervision, Methodology, Investigation, Formal analysis, Data curation. **Yeshambel Worku Demlie:** Writing – review & editing, Visualization, Resources, Methodology, Investigation, Funding acquisition, Formal analysis, Data curation. **Michael Getie:** Resources, Methodology, Investigation, Formal analysis, Data curation. **Negga Asamene Abera:** Writing – review & editing, Supervision, Resources, Investigation, Formal analysis, Data curation. **Gemechu Tadesse Leta:** Visualization, Validation, Project administration, Funding acquisition, Conceptualization. **Chalie Mulu Belete:** Validation, Software, Methodology, Investigation, Data curation. **Getachew Tollera Eticha:** Writing – review & editing, Visualization, Validation, Supervision, Project administration. **Aklilu Feleke Haile:** Writing – review & editing, Visualization, Validation, Supervision, Resources, Methodology, Investigation, Funding acquisition, Conceptualization. **Tadesse Eguale:** Writing – review & editing, Visualization, Validation, Supervision, Investigation, Formal analysis, Data curation, Conceptualization.

## Declaration of competing interest

The authors have no competing interests to declare.
